# Knock-in of the *Wt1* R394W mutation causes MDS and cooperates with *Flt3/ITD* to drive aggressive myeloid neoplasms in mice

**DOI:** 10.18632/oncotarget.26238

**Published:** 2018-10-19

**Authors:** Colleen E. Annesley, Cara Rabik, Amy S. Duffield, Rachel E. Rau, Daniel Magoon, Li Li, Vicki Huff, Donald Small, David M. Loeb, Patrick Brown

**Affiliations:** ^1^ Department of Pediatrics, University of Washington, Seattle, WA, USA; ^2^ The Sidney Kimmel Comprehensive Cancer Center at Johns Hopkins, Johns Hopkins University School of Medicine, Baltimore, MD, USA; ^3^ Department of Pediatrics, Johns Hopkins University School of Medicine, Baltimore, MD, USA; ^4^ Department of Pathology, Johns Hopkins University School of Medicine, Baltimore, MD, USA; ^5^ Department of Pediatrics, Baylor College of Medicine, Houston, TX, USA; ^6^ Department of Molecular Genetics/Cancer Genetics, University of Texas M.D. Anderson Cancer Center, Houston, TX, USA; ^7^ Current affiliation: Departments of Pediatrics and Developmental and Molecular Biology, Albert Einstein College of Medicine, Bronx, NY, USA

**Keywords:** WT1, Wilms tumor 1, myelodysplastic syndrome, AML, FLT3

## Abstract

Wilms tumor 1 (WT1) is a zinc finger transcriptional regulator, and has been implicated as both a tumor suppressor and oncogene in various malignancies. Mutations in the DNA-binding domain of the *WT1* gene are described in 10–15% of normal-karyotype AML (NK-AML) in pediatric and adult patients. Similar *WT1* mutations have been reported in adult patients with myelodysplastic syndrome (MDS). *WT1* mutations have been independently associated with treatment failure and poor prognosis in NK-AML. Internal tandem duplication (ITD) mutations of FMS-like tyrosine kinase 3 (*FLT3*) commonly co-occur with *WT1*-mutant AML, suggesting a cooperative role in leukemogenesis. The functional role of *WT1* mutations in hematologic malignancies appears to be complex and is not yet fully elucidated. Here, we describe the hematologic phenotype of a knock-in mouse model of a *Wt1* mutation (R394W), described in cases of human leukemia. We show that *Wt1*^+/R394W^ mice develop MDS which becomes 100% penetrant in a transplant model, exhibit an aberrant expansion of myeloid progenitor cells, and demonstrate enhanced self-renewal of hematopoietic progenitor cells *in vitro*. We crossbred *Wt1*^+/R394W^ mice with knock-in *Flt3*^+/ITD^ mice, and show that mice with both mutations (*Flt3*^+/ITD^/*Wt1*^+/R394W^) develop a transplantable MDS/MPN, with more aggressive features compared to either single mutant mouse model.

## INTRODUCTION

The Wilms tumor 1 gene (*WT1)* encodes a zinc finger transcriptional regulator that acts as a tumor suppressor in various cell types, with target genes implicated in cell differentiation, apoptosis and cell cycle regulation [1, 2]. Discerning the role of *WT1* in hematopoietic malignancies has proven to be complex, with conflicting reports of the prognostic value of *WT1* expression and its possible role as an oncogene [3–7]. The first evidence that a *WT1* mutation could be leukemogenic was a report describing a Wilms tumor survivor with WAGR syndrome, who by definition harbored a germline heterozygous deletion of the *WT1* gene, and later developed acute myeloid leukemia (AML) with a new somatic *WT1* mutation in the remaining allele [8].

Multiple reports have since shown that somatic *WT1* mutations are present in approximately 10–15% of both adult [9, 10] and pediatric [11, 12] patients with normal-karyotype (NK)-AML. Clusters of mutational “hot spots” occur in exons 7 and 9, which encode the zinc finger DNA-binding domain. Mutations in exon 7 tend to be frameshift mutations and often occur as biallelic compound heterozygous mutations, resulting in a truncated WT1 protein and loss of the zinc finger DNA-binding domain [9, 11]. Exon 9 mutations are often missense mutations, believed to act in a dominant negative manner, interfering with the function of wild type WT1 [13]. *WT1* mutations have also been reported in 3–4% of myelodysplastic syndrome (MDS) [14] and have been associated with an increased risk of transformation to AML [15]. Recent studies of large MDS cohorts have defined *WT1* mutations as an independent poor prognostic indicator [16], and have shown correlations of *WT1* mutations with lower hemoglobin levels and a higher percentage of bone marrow blasts [17].

*WT1* mutations frequently coexist with *FLT3*/ITD mutations in AML, suggesting possible leukemogenic cooperativity. Even when accounting for the poor prognostic implication of *FLT3*/ITD mutations, *WT1* mutations have been independently associated with treatment failure and a poor prognosis [10, 18–20]. The largest and most recent of these reports demonstrated that *WT1* mutations have an independent adverse impact on event free survival (EFS) in adults with NK-AML [20]. In pediatric AML, *WT1* mutations were also found to independently confer a poor prognosis and a higher cumulative incidence of relapse [11].

For the present study, we sought to investigate the *in vivo* effects of a *Wt1* mutation in a mouse model and describe the hematologic phenotype. We obtained mice heterozygous for the R394W mutation, the result of a C to T transition in exon 9 in the DNA-binding domain [21]. R394W has been reported in cases of human AML [22]. We found that *Wt1*^+/R394W^ mice develop MDS, which is recapitulated with 100% penetrance in a transplant mouse model at a shortened latency. To specifically investigate the relationship between *WT1* mutations and *FLT3*/ITD mutations in human AML, we created a novel mouse model by cross-breeding mice with a constitutively knocked-in *Flt3*/ITD mutation with *Wt1*^+/R394W^ mice. *Flt3*^+/ITD^ mice are well characterized and develop a fatal myeloproliferative neoplasm (MPN) [23]. We show that mice harboring both *Wt1*/R394W and *Flt3*/ITD mutations develop an aggressive MDS/MPN phenotype. Using these models, we identified a functional role for a *WT1* mutation in myeloid neoplasms.

## RESULTS

### Wt1*^+/R394W^* mice develop late-onset myelodysplastic syndrome

To investigate the effects of a *WT1* mutation on the hematopoietic system *in vivo*, we utilized a previously described knock-in *Wt1* mutant mouse model, containing the germline heterozygous mutation R394W (*Wt1*^+/R394W^) [21]. R394W is located in the DNA-binding domain of WT1 and is the most common mutation described in Denys-Drash syndrome (DDS). R394W has also been reported in cases of human leukemia [22]. Gao *et al*. reported that R394W knock-in mice develop DDS in a strain-specific background [21]. We utilized a mixed strain *Wt1*^+/R394W^ mouse model that does not develop DDS in order to study the functional effects of R394W on hematopoiesis.

**Figure 1:**
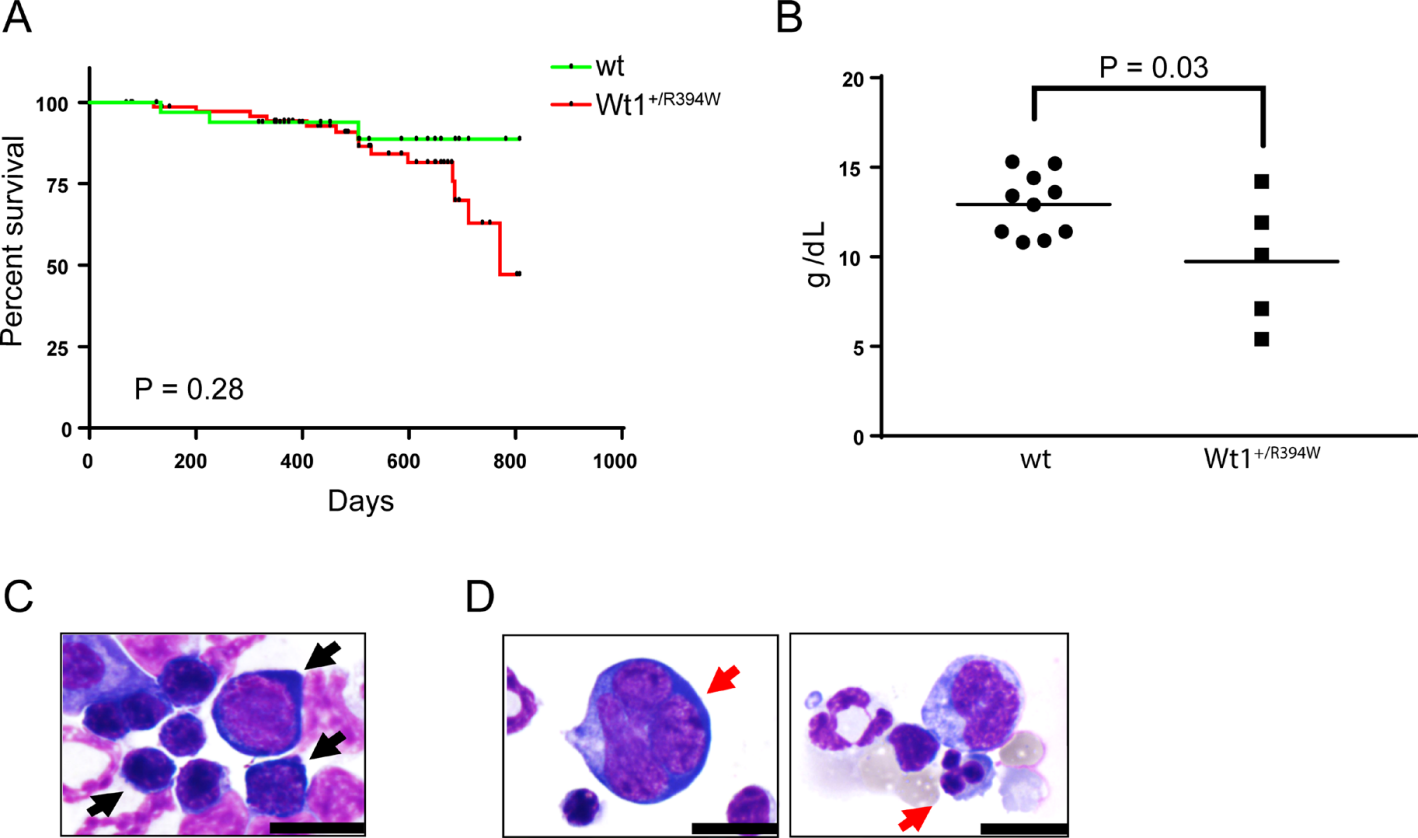
Hematologic characterization of knock-in *Wt1*^+/R394W^ mice (**A**) Kaplan–Meier survival curve of knock-in *Wt1*^+/R394W^ mice (*n* = 76) and wild type (wt) littermates (*n* = 36). (**B**) Hemoglobin values of moribund *Wt1*^+/R394W^ mice (*n* = 5, mean hemoglobin 9.74 g/dL ± 1.59) compared to age-matched wt mice (*n* = 10, mean hemoglobin 12.93 g/dL ± 0.55, *p* = 0.03). Horizontal bars represent the mean values. Representative bone marrow cytospins are shown of (**C**) wild type mice and (**D**) moribund *Wt1*^+/R394W^ mice (100×; scale bars are 10 microns). (C) Bone marrow from wt mice demonstrates no significant dysplasia in the myeloid or erythroid lineages; black arrows indicate normal erythroid precursors. In (D), red arrows denote early and late erythroid precursors with dysplastic changes in *Wt1*^+/R394W^ bone marrow.

We hypothesized that *Wt1*^+/R394W^ mice would develop an abnormal hematologic phenotype over time. We found no impact on the early survival of these mice, but observed a trend towards an inferior late survival compared to wild type littermates (Figure 1A). Multiple *Wt1*^+/R394W^ mice were found dead at an older age, and therefore we did not fully analyze all mice. However, we observed that several older *Wt1*^+/R394W^ mice appeared quite ill, and we evaluated the hematologic parameters of six moribund *Wt1*^+/R394W^ mice. Three of 5 moribund *Wt1*^+/R394W^ mice with available CBC data were anemic, compared to a cohort of age-matched wild type mice (mean hemoglobin of the five *Wt1*^+/R394W^ mice, 9.7 ± 1.6 g/dL vs. wild type mice, 12.9 ± 0.5 g/dL, *p* = 0.03; Figure 1B). Other CBC parameters (WBC, platelet counts) from the moribund *Wt1*^+/R394W^ mice did not show significant differences compared to the wild type counterparts (data not shown). To confirm that the observed anemia in the *Wt1*^+/R394W^ mice was of a purely hematologic etiology and not due to renal disease, we measured erythropoietin levels in these mice and did not observe a difference when comparing wild type and *Wt1*^+/R394W^ mice, but did observe an appropriate increase in erythropoietin in anemic mice, regardless of genotype (Supplementary Figure 1). We conclude that the anemia in the *Wt1*^+/R394W^ mice was of a primary hematologic etiology.

Morphologic examination of the bone marrow of wild type mice demonstrates normal trilineage hematopoiesis (Figure 1C). In contrast, the bone marrow of the moribund *Wt1*^+/R394W^ mice demonstrates dysplastic changes limited to the erythroid lineage, including multinucleation and irregular nuclear borders (Figure 1D). Further, *Wt1*^+/R394W^ mice demonstrate a trend toward a decreased myeloid to erythroid (M:E) cell ratio in the bone marrow compared to wild type mice (mean M:E, 2.1 ± 0.29 vs. 2.8 ± 0.26 for wild type, *p* = 0.14; Supplementary Figure 2). A limited flow cytometry panel performed on *Wt1*^+/R394W^ bone marrow and splenocytes demonstrated no abnormal population (data not shown). Thus, *Wt1*^+/R394W^ mice, with incomplete penetrance, develop a primary marrow disorder that closely recapitulates MDS with single lineage dysplasia (formerly refractory anemia) whereas the vast majority of age-matched wild type controls have no evidence of marrow disease. None of the knock-in *Wt1*^+/R394W^ mice developed overt leukemia.

### Wt1*^+/R394W^* bone marrow demonstrates expansion of hematopoietic progenitor cells

**Figure 2:**
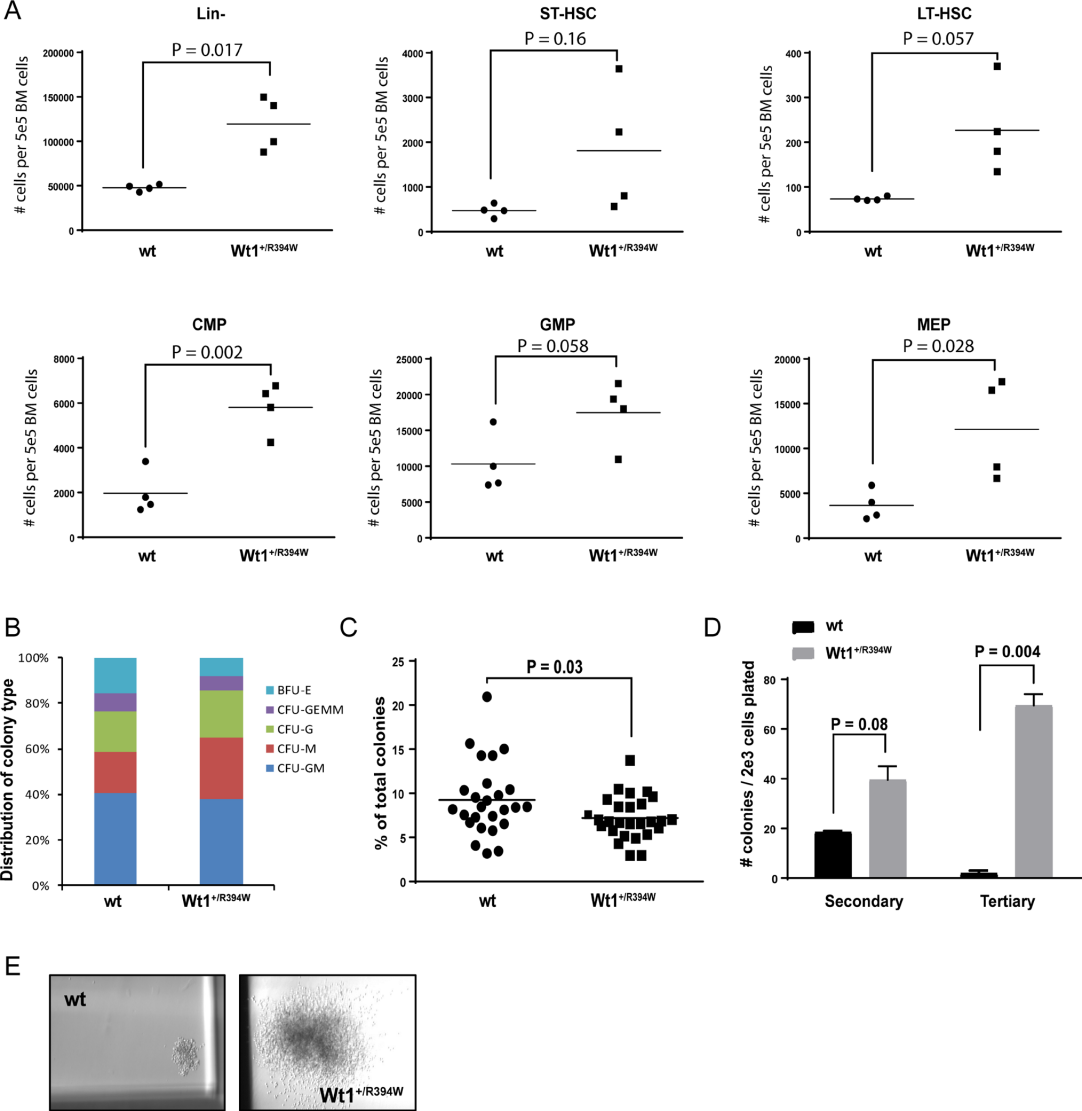
*In vitro* progenitor cell analysis of 2-month old wild type (wt) and *Wt1*^+/R394W^ bone marrow (**A**) Absolute number of cells in each progenitor cell compartment per 5 × 10^5^ live bone marrow cells of wt or *Wt1*^+/R394W^ mice (*n* = 4 each), as analyzed by flow cytometry. Short-term hematopoietic stem cells (ST-HSCs) were defined as lineage (Lin)-sca1+ckit+ (LSK) and CD34+135–; long-term (LT)-HSCs as LSK and CD34–135–; common myeloid progenitors (CMP) as Lin-sca-ckit+CD34+FcγR-; granulocyte-monocyte progenitors (GMP) as Lin-sca-ckit+CD34+FcγR+; and megakaryocyte-erythroid progenitors (MEP) as Lin-sca-ckit+CD34-FcγR–. (**B**) Distribution of colony type formation in methylcellulose culture at Day 7 after initial plating. Lineage-depleted bone marrow cells from 2-month old mice were originally plated at 2 × 10^3^ cells per mL of methylcellulose, in triplicate. Results are representative of nine separate experiments. BFU-E = burst-forming unit-erythroid; CFU-GEMM = colony-forming unit-granulocyte, erythrocyte, monocyte/macrophage, megakaryocyte; CFU-G = colony-forming unit-granulocyte; CFU-M = colony-forming unit-macrophage; CFU-GM = colony-forming unit-granulocyte/macrophage. (**C**) Number of BFU-E colonies generated from *Wt1*^+/R394W^ lineage-depleted bone marrow compared to wt bone marrow at Day 7. (**D**) Colony counts after secondary and tertiary re-plating of *Wt1*^+/R394W^ or wt bone marrow cells in methylcellulose culture, done in triplicate. Cells were harvested and re-plated every 10–12 days. (**E**) Representative colony size at tertiary re-plating (magnification 20×). Horizontal bars represent the mean values, error bars represent the SEM.

In order to determine if young *Wt1*^+/R394W^ mice demonstrate any early abnormalities in hematopoiesis, we evaluated cohorts of 2-month old mice. *Wt1*^+/R394W^ mice have mild splenomegaly compared to wild type mice (spleen weights, 0.11 ± 0.005 g vs. 0.09 ± 0.007 g for wild type, *p* = 0.049), but there were no appreciable differences in the peripheral blood counts between 2-month old wild type and *Wt1*^+/R394W^ littermates (Supplementary Figure 3). In the bone marrow, we observe an overall increase in the number of progenitor cells in *Wt1*^+/R394W^ mice, with a statistically significant increase in the common myeloid progenitor (CMP; mean absolute number of CMPs per 5 × 10^5^ bone marrow cells, 5811 ± 560 vs. 1973 ± 486 for wild type, *p* = 0.002) and megakaryocyte-erythroid progenitor (MEP; mean absolute number of MEPs per 5 × 10^5^ bone marrow cells, 12143 **±** 2809 vs. 3659 **±** 839 for wild type, *p* = 0.028) compartments (Figure 2A). Flow cytometry did not reveal any abnormalities in the differentiated cell populations in the bone marrow (data not shown). In methylcellulose colony-forming assays, lineage-depleted bone marrow cells from 2-month old *Wt1*^+/R394W^ mice demonstrate a significant decrease in the number of erythroid colonies (BFU-E) formed compared to wild type (Figure 2B–2C). Together, these data indicate that subgroups of *Wt1*^+/R394W^ progenitor cells are aberrantly expanded in number *in vivo*, and demonstrate ineffective erythropoiesis *in vitro*.

We also found that *Wt*1^+/R394W^ progenitor cells possess significantly increased serial re-plating capacity, with markedly increased colony number and size at tertiary plating (mean colony number, 69.5 vs. 2 for wild type, *p* = 0.004; Figure 2D–2E). These data suggest that the *Wt1* R394W mutation leads to aberrant self-renewal capacity *in vitro,* indicative of transformative potential.

### Wt1*^+/R394W^* megakaryocyte-erythroid progenitor cells demonstrate a functional disadvantage in a competitive transplant model

**Figure 3:**
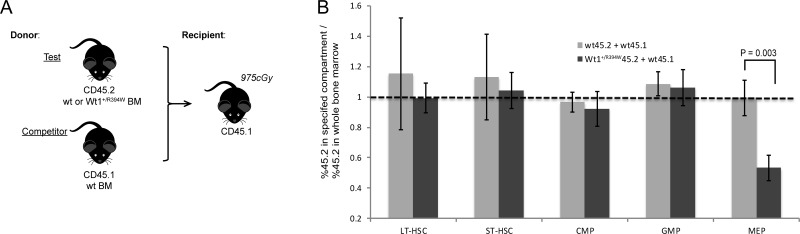
Competitive bone marrow transplant with wild type (wt) and *Wt1*^+/R394W^ progenitor cells (**A**) Competitive transplant schema. *N* = 5 mice in each test group (wt or *Wt1*^+/R394W^ CD45.2 cells). (**B**) Engraftment contribution of CD45.2 bone marrow to each individual progenitor cell compartment at 16 weeks after transplant. Ratio of 1 (dotted horizontal line) defines an equal contribution of CD45.2 cells to that compartment normalized to the amount of CD45.2 cells in the total bone marrow. (ST-HSCs = short-term hematopoietic stem cells, LT-HSCs = long-term hematopoietic stem cells, CM*P* = common myeloid progenitors, GM*P* = granulocyte-monocyte progenitors, and ME*P* = megakaryocyte-erythroid progenitors). Error bars represent the SD.

Given the enhanced self-renewal capacity of the *Wt1*^+/R394W^ stem cells *in vitro*, we sought to determine if the *Wt1* mutation confers a competitive advantage *in vivo*. Using a 1:1 mixture of wild type CD45.1 bone marrow and either wild type or *Wt1*^+/R394W^ CD45.2 bone marrow (Figure 3A), cells were transplanted into lethally irradiated CD45.1 recipients (*n* = 5 recipient mice/each). As expected, we observe an average of roughly 50% engraftment of CD45.2 wild type cells at both 4 and 12 weeks, as measured by peripheral blood chimerism (Supplementary Figure 4A). Interestingly, despite an early engraftment disadvantage (less than 50% at 4 weeks), the *Wt1*^+/R394W^ CD45.2 cells demonstrate improved engraftment in the peripheral blood at 12 weeks post-transplant.

Sixteen weeks after transplant, we sacrificed the mice and evaluated the engraftment contribution of CD45.2 cells in the bone marrow. We found no statistically significant competitive advantage of the CD45.2 *Wt1*^+/R394W^ cells in the bone marrow (mean CD45.2%, 63.5 ± 12.6% for *Wt1*^+/R394W^ vs. 53.5 ± 10.2% for wild type; *p* = 0.21; Supplementary Figure 4B). Therefore, although the R394W *Wt1* mutation clearly leads to increased progenitor cell self-renewal *in vitro* (Figure 2D–2E), it does not confer a fully proliferative competitive advantage *in vivo*. We next evaluated the relative engraftment contribution of CD45.2 bone marrow to each individual progenitor cell compartment (Figure 3B). A ratio of 1 suggests an equal contribution of CD45.2 cells to that compartment, normalized to the amount of CD45.2 cells in the total bone marrow. In the control group that received wild type CD45.2 donor bone marrow, all compartments have an equal relative engraftment of CD45.2. Interestingly, the *Wt1*^+/R394W^ CD45.2 bone marrow exhibit a statistically significant, relative engraftment disadvantage in the MEP compartment (mean ratio 0.53 ± 0.08 vs. 1.0 **±** 0.12 for wild type, *p* = 0.003). Thus, although the MEPs are numerically expanded in the bone marrow of *Wt1*^+/R394W^ mice (Figure 2A), these competitive repopulation data suggest that the *Wt1*^+/R394W^ MEPs are functionally abnormal, complementary to our finding that *Wt1*^+/R394W^ mice develop MDS characterized by dyserythropoiesis and anemia.

### Wt1*^+/R394W^* mice with concomitant Flt3/ITD mutations develop MDS/MPN

**Figure 4:**
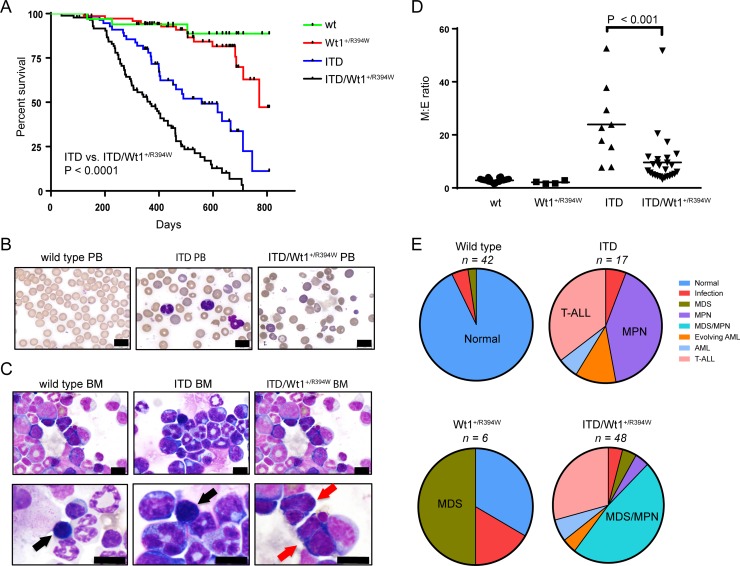
Hematologic characterization of a crossbred *Wt1*^+/R394W^ x *Flt3*^+/ITD^ mouse model (**A**) Kaplan–Meier survival curve of the crossbred mice containing both mutations (ITD/*Wt1*^+/R394W^) compared to mice with either mutation alone (*Wt1*^+/R394W^, *p* < 0.0001 or ITD, *p* < 0.0001) and compared to wild type (wt) littermates. (**B**) Representative peripheral blood smears from wt, ITD, and ITD/*Wt1*^+/R394W^ mice(40×; scale bars are 10 microns). (**C**) Representative bone marrow cytospins from wt, ITD, and ITD/*Wt1*^+/R394W^mice. Black arrows point to normal erythroid precursors, and red arrows indicate erythroid precursors with dysplastic changes in the ITD/*Wt1*^+/R394W^ mice (top row 40×, bottom row 100×; all scale bars are 10 microns). (**D**) Comparison of myeloid to erythroid (M:E) ratios in the bone marrow of each genotype. Mean values for each genotype: wt 2.89 ± 0.12 (*n* = 40), *Wt1*^+/R934W^ 2.13 ± 0.29 (*n* = 4), ITD 32.46 ± 5.94 (*n* = 12), ITD/*Wt1*^+/R394W^ 10.11 ± 1.67 (*n* = 32). Horizontal bars represent the mean values, error bars represent the SEM. (**E**) Distribution of hematologic phenotype by mouse genotype. Phenotype definitions are based on the Bethesda classification, and the predominant phenotype in each genotype is highlighted. MDS = myelodysplastic syndrome, MPN = myeloproliferative neoplasm, AML = acute myeloid leukemia, T-ALL = T-cell acute lymphoblastic leukemia.

Patients with AML and *WT1* mutations frequently also harbor *FLT3*/ITD mutations, suggesting cooperative leukemogenesis [9, 11]. Therefore, we utilized a previously described knock-in mouse model heterozygous for a *Flt3*/ITD mutation (*Flt3*^+/ITD^), and crossbred these with *Wt1*^+/R394W^ mice. *Flt3*^+/ITD^ mice develop a fatal myeloproliferative neoplasm (MPN) at a mean latency of 10 months, but do not independently develop myeloid leukemia [23]. In other *Flt3*/ITD cooperative models, both myeloid and lymphoid leukemic phenotypes have been observed [24, 25]. Here, we found that mice harboring both mutations (*Flt3*^+/ITD^/*Wt1*^+/R394W^) have an inferior survival compared to *Flt3*^+/ITD^ mice (*p* < 0.001; Figure 4A). By flow cytometry analysis, *Flt3*^+/ITD^/*Wt1*^+/R394W^ mice develop either an MPN-like disease or T cell acute lymphoblastic leukemia (T-ALL; not shown) similar to *Flt3*^+/ITD^ mice previously described, although with a shorter average latency. One of 17 evaluated *Flt3*^+/ITD^ mice did develop AML, though this mouse was found to have loss of heterozygosity (LOH) of the wild type *Flt3* allele (Supplementary Figure 5A). This phenomenon of LOH of wild type *FLT3* has previously been described, and causes a more aggressive disease phenotype in both humans and mice [24, 26, 27]. Three of the *Flt3*^+/ITD^/*Wt1*^+/R394W^ mice developed AML, one of which had LOH of wild type *Flt3*.

Although 35% (6/17) of *Flt3*^+/ITD^ mice and 29% (14/48) of *Flt3*^+/ITD^/*Wt1*^+/R394W^ mice developed T-ALL, we were specifically interested in studying the impact of these mutations on myeloid development. Analysis of mice that died of myeloid disease shows that *Flt3*^+/ITD^/*Wt1*^+/R394W^ mice have lower median hemoglobin values compared to mice with either mutation alone, though these differences are not statistically significant (Supplementary Figure 6). In the peripheral blood, *Flt3*^+/ITD^ mice have circulating atypical monocytes (as previously described) [23] and red blood cell (RBC) polychromasia, suggesting increased premature release of RBCs from the marrow (Figure 4B). *Flt3*^+/ITD^/*Wt1*^+/R394W^ mice also demonstrate RBC polychromasia in the peripheral blood, but have more pronounced RBC fragmentation and anisopoikilocytosis (Figure 4B). The bone marrow morphology of *Flt3*^+/ITD^ mice demonstrates a marked myeloid predominance compared to wild type bone marrow, with a relatively decreased number of erythroid precursors, consistent with an MPN (Figure 4C). Though few in number, the erythroid precursors in the *Flt3*^+/ITD^ bone marrow show no overt dysplasia. In striking contrast, *Flt3*^+/ITD^/*Wt1*^+/R394W^ mice exhibit dysplastic changes in the erythroid lineage with irregular nuclear borders and multinucleate erythroid precursors reminiscent of the *Wt1*^+/R394W^ mice (Figure 4C), though with myeloid proliferation reflective of the ITD-induced MPN. The findings of both dysplastic and proliferative features in the *Flt3*^+/ITD^/*Wt1*^+/R394W^ mice are compatible with a myelodysplastic disorder/myeloproliferative neoplasm (MDS/MPN) [28].

Further, we noted a relative increase in the number of erythroid precursors in the bone marrow of *Flt3*^+/ITD^/*Wt1*^+/R394W^ mice compared to the *Flt3*^+/ITD^ mice. *Flt3*^+/ITD^ mice have very high M:E ratios due to the proliferation of myeloid precursors, and the highest M:E ratios were seen in mice with ITD mutations and LOH of wild type *Flt3*. In contrast, *Flt3*^+/ITD^/*Wt1*^+/R394W^ mice have relatively lower M:E ratios (mean M:E ratio, 10.11 ± 1.67 vs. 32.46 ± 5.94 for *Flt3*^+/ITD^; Figure 4D), consistent with the increased erythroid precursors also seen in the *Wt1*^+/R394W^ mice. In comparing the small numbers of mice with *Flt3* LOH, the M:E ratios are again lower in the *Flt3*^+/ITD^/*Wt1*^+/R394W^ mice compared to *Flt3*^+/ITD^ mice (Supplementary Figure 5B). Therefore, each genotype is associated with characteristic hematologic phenotypes (Figure 4E, Table 1). The vast majority of wild type mice have normal bone marrow, the majority of *Flt3*^+/ITD^ mice develop a myeloid neoplasm (primarily MPN), the predominant phenotype in the *Wt1*^+/R394W^ mice evaluated is MDS, and the majority of *Flt3*^+/ITD^/*Wt1*^+/R394W^ mice develop an MDS/MPN.

**Table 1:** Distribution of hematologic phenotype by mouse genotype

Disease Phenotype	Wild type (*n* = 42)	Wt1^+/R394W^ (*n* = 6)	Flt3/^+/ITD^ (*n* = 17)	Flt3/^+/ITD^/Wt1^+/R394W^ (*n* = 48)
Normal	39 (93%)	2 (33%)	0 (0%)	0 (0%)
Infection	2 (5%)	1 (17%)	1 (6%)	2 (4%)
MDS	1 (2%)	3 (50%)	0 (0%)	2 (4%)
MPN	0 (0%)	0 (0%)	7 (41%)	2 (4%)
MDS/MPN	0 (0%)	0 (0%)	0 (0%)	23 (48%)
Evolving AML	0 (0%)	0 (0%)	2 (12%)	2 (4%)
AML	0 (0%)	0 (0%)	1 (6%)	3 (6%)
T-ALL	0 (0%)	0 (0%)	6 (35%)	14 (29%)

Abbreviations: MDS = myelodysplastic syndrome, MPN = myeloproliferative neoplasm, AML = acute myeloid leukemia, T-ALL = T-cell acute lymphoblastic leukemia.

### Transplant model of Wt1*^+/R394W^* accelerates and enhances MDS phenotype

**Figure 5:**
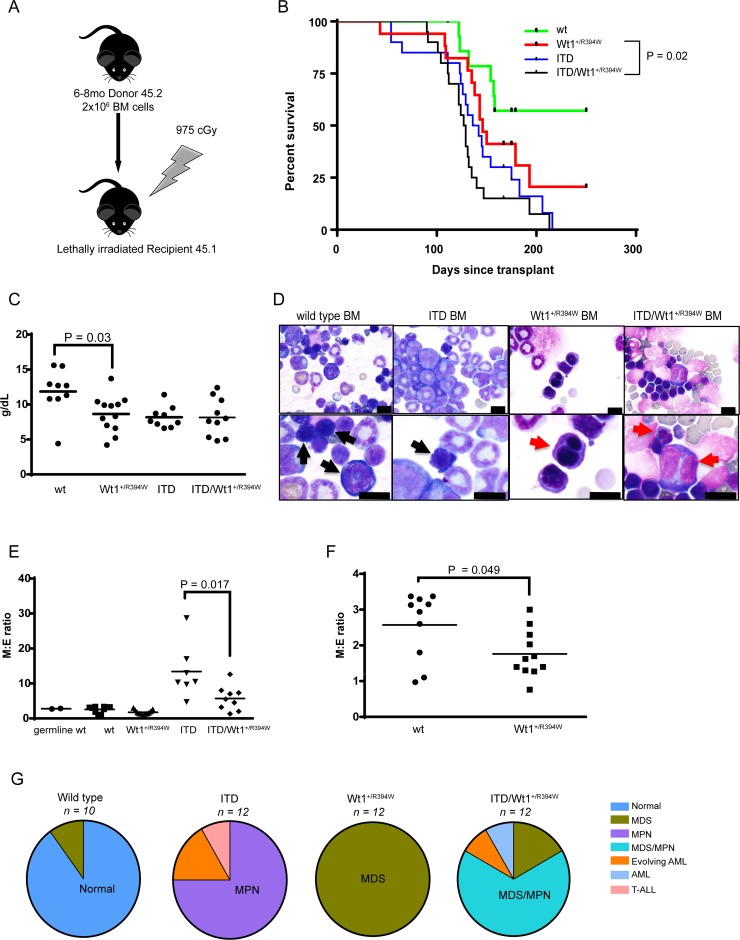
Hematologic characterization of a transplant model of *Wt1*^+/R394W^ x *Flt3*^+/ITD^ (**A**) Schema of creation of transplant model. Donor mice were aged 6–8 months. (**B**) Kaplan–Meier survival curve of the transplanted mice: recipients of bone marrow with both mutations (ITD/*Wt1*^+/R394W^), either mutation alone (*Wt1*^+/R394W^ or ITD), or wild type (wt). Survival is measured as days post-transplant; *p* = 0.2 for ITD vs. ITD/*Wt1*^+/R394W^; p=0.02 for *Wt1*^+/R394W^ vs. ITD/*Wt1*^+/R394W^; p = 0.14 for *Wt1*^+/R394W^ vs. wt. (**C**) Hemoglobin values of transplanted moribund mice of each bone marrow genotype at time of sacrifice. Horizontal bars represent the mean hemoglobin value for each genotype: wt 11.86 ± 1.11 g/dL, *Wt1*^+/R394W^ 8.64 ± 0.76 g/dL, ITD 8.18 ± 0.51 g/dL, ITD/*Wt1*^+/R394W^ 8.14 ± 0.86 g/dL. (**D**) Representative bone marrow cytospins from wt, ITD, *Wt1*^+/R394W^ and ITD/*Wt1*^+/R394W^ recipient mice. Black arrows indicate normal erythroid precursors in the wt and ITD bone marrows, and red arrows point to the dysplastic erythroid precursors in the Wt1+/R394W and ITD/*Wt1*^+/R394W^ bone marrows (top row 40×, bottom row 100×; all scale bars are 10 microns). (**E**) Comparison of myeloid to erythroid (M:E) ratios in the bone marrow. Mean values: germ line wt 2.78 ± 0.11; transplanted wt 2.57 ± 0.3, *Wt1*^+/R394W^ 1.76 ± 0.2, ITD 13.41 ± 2.91, ITD/*Wt1*^+/R394W^ 5.71 ± 1.17. (**F**) Zoomed view comparison of M:E ratios in *Wt1*^+/R394W^ versus wt transplanted bone marrow. Horizontal bars represent the mean value. (**G**) Distribution of hematologic phenotype by bone marrow genotype of the transplanted mice. The predominant phenotype in each genotype is highlighted. MDS = myelodysplastic syndrome, MPN = myeloproliferative neoplasm, AML = acute myeloid leukemia, T-ALL = T-cell acute lymphoblastic leukemia.

To isolate the hematopoietic effects of the mutations, we transplanted pre-disease bone marrow from six-to-eight month old mice from each genotype into lethally irradiated congenic CD45.1 mice (Figure 5A). The *Flt3*^+/ITD^/*Wt1*^+/R394W^ bone marrow recipients demonstrate inferior survival compared to *Wt1*^+/R394W^ recipients (*p* = 0.02), and the *Wt1*^+/R394W^ bone marrow recipients show a trend toward inferior survival compared to wild type recipients (Figure 5B). Recipients of wild type bone marrow also have a shortened survival in this model, though in the majority of cases, there was no abnormal hematological phenotype found. We hypothesize the wild type marrow recipients may have eventually died from late radiation effects, although among other possibilities is graft versus host disease.

This transplant model recapitulates the phenotypes observed in the germline models of these mutations, with a shorter latency to disease onset. Anemia is observed in recipients of all abnormal marrow genotypes, but not in wild type bone marrow recipients (mean hemoglobin, 8.64 ± 0.76 g/dL for *Wt1*^+/R394W^ recipients, vs. 11.86 ± 1.11 g/dL for wild type, *p* = 0.03; Figure 5C). Morphologic examination of the bone marrow demonstrates erythrodysplasia in all *Wt1*^+/R394W^ recipient mice, myeloid proliferation in the majority of the *Flt3*^+/ITD^ recipient mice, and features of both MDS and MPN in the majority of the *Flt3*^+/ITD^/*Wt1*^+/R394W^ recipient mice (Figure 5D). T-ALL in recipients of ITD mutant bone marrow is rare in this transplant model. We determined the M:E ratios of these transplanted mice, and observe a similar pattern similar to that seen in the germline model (Figure 5E–5F). Therefore, the predominant hematologic phenotypes are the same in this transplant model as compared to the germline mutant mice (Figure 5G, Table 2). Strikingly, in this model, 100% of the evaluable *Wt1*^+/R394W^ marrow transplant recipients develop MDS. These data demonstrate that the *Wt1* R394W mutation consistently produces transplantable myeloid neoplasms in a mouse model.

**Table 2:** Distribution of hematologic phenotype by bone marrow genotype of transplanted mice

Disease Phenotype	Wild type BM (*n* = 10)	Wt1^+/R394W^ BM (*n* = 12)	Flt3/^+/ITD^ BM (*n* = 12)	Flt3/^+/ITD^/Wt1^+/R394W^ BM (*n* = 12)
Normal	9 (90%)	0 (0%)	0 (0%)	0 (0%)
Infection	0 (0%)	0 (0%)	0 (0%)	0 (0%)
MDS	1 (10%)	12 (100%)	0 (0%)	2 (17%)
MPN	0 (0%)	0 (0%)	9 (75%)	0 (0%)
MDS/MPN	0 (0%)	0 (0%)	0 (0%)	8 (67%)
Evolving AML	0 (0%)	0 (0%)	2 (17%)	1 (8%)
AML	0 (0%)	0 (0%)	0 (0%)	1 (8%)
T-ALL	0 (0%)	0 (0%)	1 (8%)	0 (0%)

Abbreviations: BM = bone marrow, MDS = myelodysplastic syndrome, MPN = myeloproliferative neoplasm, AML = acute myeloid leukemia, T-ALL = T-cell acute lymphoblastic leukemia.

## DISCUSSION

Multiple reports have shown that *WT1* mutations are a recurrent event in AML and MDS, yet it is not well understood how *WT1* mutations contribute to the development of hematologic malignancies. In the present study, we show that mice heterozygous for a knocked-in *Wt1* R394W mutation can develop anemia with concomitant erythroid dysplasia, consistent with ineffective erythropoiesis and MDS. In human disease, MDS limited to the erythroid series often does not show overt phenotypic abnormalities in a limited flow panel; therefore, the normal flow cytometry analysis of bone marrow from moribund *Wt1^+/R394W^* mice with MDS in our series was not unexpected. We found that *Wt1*^+/R394W^ mice demonstrate disordered hematopoiesis as young as two months of age, as evidenced by an abnormal quantity and quality of myeloid progenitor cell compartments, and *Wt1*^+/R394W^ hematopoietic progenitor cells demonstrate aberrant self-renewal behavior *in vitro*. Interestingly, in a competitive *in vivo* model, *Wt1* mutant cells exhibited a relative repopulation disadvantage, specific to the MEP compartment. We found that marrow reconstitution with a heterozygous *Wt1* R394W in a lethally irradiated transplant mouse model results in the development of MDS with a shortened latency and with 100% penetrance, whereas not all mice in the germline model developed MDS. Conversely, only one of 10 mice (10%) transplanted with wild type bone marrow developed signs of MDS. This is compelling evidence that the *Wt1* R394W mutation is sufficient to cause MDS.

Further, when we crossed *Flt3*^+/ITD^ mice with *Wt1*^+/R394W^ mice, the majority of double mutant offspring developed an MDS/MPN. These mice displayed disease manifestations of shortened survival, myeloid expansion in the bone marrow (exaggerated in cases of LOH of wild type *Flt3*), anemia, erythroid dysplasia, and a relatively decreased M:E ratio compared to the *Flt3*^+/ITD^ mice. Although coincident *Flt3*/ITD and *Wt1*/R394W mutations do not appear sufficient to consistently recapitulate human AML in a mouse model, our results demonstrate that *Flt3*/ITD and *Wt1*/R394W mutations are capable of cooperation, resulting in a more aggressive disease than resulted from either individual mutant genotype alone. Of interest, a small number of mice with *Flt3*/ITD with or without a *Wt1* mutation did develop AML, suggesting that an additional acquired “hit” may have contributed to leukemogenesis in these mice.

Previous mouse models have examined the effect of loss of *Wt1* on murine hematopoiesis. Chimeric mice generated from a mixture of normal blastocysts and embryonic stem cells with homozygous loss of *Wt1* showed that cells lacking *Wt1* did not contribute to the hematopoietic system in a competitive environment [29]; an interesting parallel to the relative lack of contribution of *Wt1*^+/R394W^ bone marrow cells to the MEP compartment in our competitive repopulation model. Another study found that *Wt1* null fetal liver cells could normally repopulate the hematopoietic system in lethally irradiated adult mice in the absence of competition, and found no difference in *in vitro* colony forming capacity between wild type and *Wt1* null cells [30]. Finally, a recent study demonstrated that a subset of aged *Wt1*-haploinsufficient mice developed features of myeloproliferation and myelodysplasia, including hepatomegaly with CD45+/Mac1+/Gr1+ infiltration, and megakaryocyte dysplasia in the bone marrow [31]. Further, a subset of secondary transplant recipients of *Wt1*-haploinsufficent bone marrow developed T-ALL after acquiring additional mutations in known leukemic alleles such as *Notch1*. In comparison, our data supports that the R394W *Wt1* mutation behaves in a dominant fashion to induce dyserythropoiesis and myeloid neoplasms. Pronier *et al.* showed that C57BL/6 mice homozygous for *Flt3*/ITD and *Wt1*-haploinsufficient developed AML, whereas mice heterozygous for Flt3/ITD and *Wt1*-haploinsufficent did not develop leukemia [31]. In comparison, a subset of our *Flt3*^+/ITD^/*Wt1*^+/R394W^ mice developed AML, including one mouse with LOH of *Flt3*. Overall, there is mounting evidence for the role of disrupted WT1 function contributing to the development of hematologic malignancies.

Interestingly, there are several striking similarities between our mouse model and *Tet2* knockout mouse models. A model of complete conditional *Tet2* loss in the hematopoietic compartment demonstrates increased stem cell self-renewal *in vitro* by serial replating studies and *in vivo* by competitive transplant studies, occurring to a lesser degree with haploinsufficient *Tet2* cells, and mice develop a phenotype resembling chronic myelomonocytic leukemia (CMML) [32]. In a knockout *Tet2* model, mice developed a variety of myeloid malignancies, including CMML, MPN, and MDS; and bone marrow showed a decreased M:E ratio with erythroid infiltration [33]. In another model, concurrent deletion of *Tet2* and *Ezh2* cooperated to cause more advanced myelodysplasia and accelerated the development of myeloid disorders including MDS/MPN [34]. These similarities are of interest to the present study, as reports of mutual exclusivity among *WT1*, *TET2* and *IDH* mutations in human AML [35] and a physical interaction between WT1 and TET2 suggest these mutations converge on a common functional pathway [36].

Our novel observations generate several hypotheses. The irregular expansion and dysfunction of pre-disease *Wt1* mutant hematopoietic MEP progenitor cells in our model, coupled with the eventual development of erythroid dysplasia and anemia in the *Wt1* mutant mouse model, indicate that a mutation in *Wt1* leads to a disease phenotype by interfering with normal erythropoiesis. Preliminary *in vitro* work from our lab suggest that *WT1* mutations can block terminal myeloid differentiation [37], and the results from our mouse model suggest *Wt1* mutations may lead to disordered erythroid maturation and differentiation. This model could also be used to further explore the functional interaction between WT1 and TET2. Evidence supports a functional relationship between WT1 and TET2, and it has been demonstrated that WT1 alters gene expression, methylation and hydroxymethylation [36, 38]. Promoter DNA methylation profiling of *WT1* mutant AML patient samples demonstrated aberrant hypermethylation compared to wild type bone marrow or AML1-ETO AML [38]. Significant overlap was seen between hypermethylated loci in *WT1*-mutant AML compared to that seen in *TET2*- and *IDH1/2*-mutant AML patient samples, further supporting a common oncogenic pathway involving these genes [38]. Rampal *et al*. also reported a global decrease in DNA hydroxymethylation (5-hmC) levels in *WT1*-, *TET2*-, and *IDH1/2*-mutant AML samples [38]. Our *Wt1*^+/R394W^ mouse model can serve as a tool to further investigate differences in DNA methylation and hydroxymethylation, both globally and at the gene level, in the setting of a leukemogenic *WT1* mutation, which has potential therapeutic implications.

There are several limitations to the current study. The knock-in germline *Wt1*^+/R394W^ mice develop MDS, but only after a long latency and with incomplete penetrance. Although this pattern does in fact recapitulate the timeline and penetrance of human disease, this model alone could make disease study challenging. Additionally, the *Wt1*^+/R394W^ germline mouse model has potential to develop Denys-Drash syndrome in a strain-dependent manner, leading to other non-hematopoietic morbidities. We did not observe clinically significant DDS in the model described here. Importantly, both of these potential limitations are addressed by utilizing a transplant model, which limits the expression of mutant *Wt1* to the hematopoietic system, and leads to increased penetrance of disease with a shortened latency. It is interesting that only 3–4% of MDS patients are reported to have *WT1* mutations, although a significant proportion of patients with MDS have *TET2* mutations, and therefore are unlikely to harbor concurrent *WT1* mutations if these mutations are linked in a common functional pathway.

In our model, roughly a third of mice with *Flt3*/ITD mutations develop T-ALL. *FLT3*/ITD mutations are observed in human T-ALL [39], and a combination of myeloid and lymphoid malignancies have previously been reported in cooperative mouse models with ITD mutations [24, 25]. However, the majority of *Flt3*^+/ITD^ mice in our model developed MPN, and the majority of *Flt3*^+/ITD^/*Wt1*^+/R394W^ mice developed MDS/MPN. In the transplant model, T-ALL was only rarely observed in mice with *Flt3*/ITD mutant bone marrow. The *Flt3*/ITD and *Wt1* mutations did not consistently cooperate to recapitulate human AML in our model; thus, a third hit is likely necessary to transform fully to leukemia. These mutations did, however, demonstrate cooperativity by resulting in inferior survival and more aggressive myeloid neoplasms.

In conclusion, we demonstrate here that the presence of *Wt1*^+/R394W^ in the murine hematopoietic system leads to the development of MDS with single lineage dysplasia, manifested as anemia and erythroid dysplasia, and contributing to a trend in decreased survival. Additionally, mice with both *Wt1*/R394W and *Flt3*/ITD mutations develop an aggressive mixed MDS/MPN. To our knowledge, this is the first characterization of a hematologic phenotype of a *WT1* mutation *in vivo*, and provides an important research tool given a relative paucity of MDS model systems.

## MATERIALS AND METHODS

### Mouse models

Mice in a mixed C57BL/6 and 129/SvEv background and heterozygous for the point mutation C1180T knocked into in exon 9 of the *Wt1* gene (encoding the amino acid change R394W), and C57BL/6 mice heterozygous for an 18-base pair ITD knocked into the juxtamembrane domain of the *Flt3* gene, had been generated previously [21, 23]. The *Wt1*^+/R394W^ mice were backcrossed to a C57BL/6 background. These two models were crossbred and offspring were characterized as wild type, positive for the *Wt1* R394W mutation (*Wt1*^+/R394W^), positive for the *Flt3*/ITD mutation (*Flt3*^+/ITD^), or positive for both mutations (*Flt3*^+/ITD^/*Wt1*^+/R394W^), based on PCR analysis of germline DNA using primers previously described [21, 23].

### Complete blood count and cytology

Complete blood cell counting was performed using the Hemavet950 (Drew Scientific). Peripheral blood smears and cytospins were visualized using a modified Wright-Giemsa stain (Sigma-Aldrich). Representative histopathology images were acquired using an Olympus BX46 microscope with an Olympus DP72 camera (at 100×). The image acquisition software used was Olympus CellSens Standard 1.5.

### Erythropoietin quantification

Erythropoietin levels from mouse serum were measured using the Quantikine**^®^** ELISA Mouse Erythropoietin Immunoassay (R&D Systems, Minneapolis, MD, USA). Briefly, mouse plasma was diluted and processed along with internal standards, adding mouse erythropoietin conjugate to wells prior to quantification on a microplate reader (Bio-Rad, Hercules, CA, USA) at 450 and 540 nm. Readings from 540 nm were subtracted from the 450 nm readings. A standard curve was generated, and sample data were plotted against the standard curve.

### *In vitro* clonogenic and serial re-plating assays

Lineage-negative bone marrow progenitor cells from 2-month-old mice were plated at 2 × 10^3^ cells/mL in methylcellulose medium supplemented with recombinant murine SCF (50 ng/mL), IL-3 (10 ng/mL), IL-6 (10 ng/mL), GM-CSF (10 ng/mL), and EPO (3U/mL) (Methocult M3434: StemCell technologies, Vancouver, BC, USA). Lineage depletion was performed as previously described, using a magnetic-column exclusion method [23]. Colonies in methylcellulose were scored 9–11 days after plating. For serial re-plating, cells were passaged in fresh methylcellulose every 10–12 days. Cells were isolated from the plates and analyzed by flow cytometry. Experiments were performed in triplicate.

### Flow cytometry

Cells were prepared and stained as previously described [24] with details in supplemental methods.

### Competitive bone marrow repopulation assay

A total of 5 × 10^5^ CD45.1^+^ competitor bone marrow cells obtained from wild type B6-Ly5.2 (CD45.1^+^ mice; National Cancer Institute and Charles River) were mixed with 5 × 10^5^ CD45.2^+^ test bone marrow cells (wild type or *Wt1*^+/R394W^ mice) and injected retroorbitally into lethally irradiated (975c Gy) B6-Ly5.2 recipient mice. Beginning at 4 weeks after transplant, peripheral blood cells were analyzed for CD45.1 and CD45.2 expression by flow cytometry.

### Transplant mouse model

Wild type CD45.1^+^ mice (National Cancer Institute) received 975cGy of gamma irradiation, followed by retroorbital injection of 2 × 10^6^ whole bone marrow cells from CD45.2^+^ wild type, *Wt1*^+/R394W^, *Flt3*^+/ITD^ or *Flt3*^+/ITD^/*Wt1*^+/R394W^ mice. Donor CD45.2^+^ mice were six to eight months of age, and donor marrow was pre-disease or had mild characteristics of early hematopoietic disease. Engraftment was evaluated by the percentage of CD45.2^+^ cells in the peripheral blood of recipient mice, as measured by flow cytometry. All animal experiments were reviewed and approved by the Johns Hopkins University Animal Care and Use Committee.

### Statistics

Data are expressed as the mean ± SEM or SD, where applicable. Unpaired *t*-tests or log-rank tests were used to compare groups (GraphPad Prism, LaJolla, CA, USA). Mantel-Cox log-rank tests were used to compare survival between groups. Values of *p* < 0.05 were considered to be significant.

## SUPPLEMENTARY MATERIALS FIGURES


