# Supplementary material for: Knock-in of the Wt1 R394W mutation causes MDS and cooperates with Flt3/ITD to drive aggressive myeloid neoplasms in mice
Source: Oncotarget. 2018 Oct 19;9(82):35313–26. doi: 10.18632/oncotarget.26238 (PMC6219680; doi:10.18632/oncotarget.26238)
Supplement: Supplementary file 1 [file oncotarget-09-35313-s001.pdf]

Knock-in of the *Wt1* R394W mutation causes MDS and cooperates with *Flt3/ITD* to drive aggressive myeloid neoplasms in mice

SUPPLEMENTARY MATERIALS

MATERIALS AND METHODS

Flow cytometry

Flow cytometric analysis of murine bone marrow, and splenocytes when indicated, were performed with the monoclonal antibodies (all from BD Pharmingen, Franklin Lakes, NJ, USA, unless otherwise specified): lineage mixture (FITC; Biolegend, San Diego, CA, USA), Ly-6A/E (Sca-1; Invitrogen), CD135 (FLT3), CD117 (c-Kit; APC), CD41, Ter-119 (PE), CD11b (Mac1; PerCP), Gr1 (APC), CD24 (FITC), CD43 (PE), CD19 (PerCP; eBioscience, ThermoFisher, Waltham, MA, USA), CD45R/B220 (APC), CD4 (FITC), CD3 (PE), CD8a (PerCP), CD34 (APC), CD16/32 (FcgR), CD45.1 (PE) and CD45.2 (APC). When performing six- or seven-color flow, the following monoclonal antibodies were also used: lineage mixture (AlexaFluor) (Biolegend), Ly-6A/E (Sca1; Cy7). Fluorescence-activated cell sorting (FACS) was performed using either a FACSCalibur or FACSAria II (BD Biosciences, San Jose, CA, USA). Analysis was performed using FlowJo version 8.8.7 (TreeStar, Ashland, OR, USA) or FACSDiva software (BD Biosciences). Murine hematopoietic stem and progenitor cell compartments were defined as previously described [1, 2]: short-term hematopoietic stem cells (ST-HSCs) as Lin-Sca1+cKit+ (LSK) CD34+CD135–; long-term (LT)-HSCs as LSK and CD34-CD135–; granulocyte-monocyte progenitors (GMP) as Lin-Sca1-cKit+CD34+FcγR+; common myeloid progenitors (CMP) as Lin-Sca1-cKit+CD34+FcγR-; and megakaryocyte-erythroid progenitors (MEP) as Lin-Sca1-cKit+CD34-FcγR-.

REFERENCES

1. Iwasaki H, Akashi K. Myeloid lineage commitment from the hematopoietic stem cell. Immunity. 2007; 26:726–740.
2. Greenblatt S, Li L, Slape C, Nguyen B, Novak R, Duffield A, Huso D, Desiderio S, Borowitz MJ, Aplan P, Small D. Knock-in of a FLT3/ITD mutation cooperates with a NUP98-HOXD13 fusion to generate acute myeloid leukemia in a mouse model. Blood. 2012; 119:2883–2894.

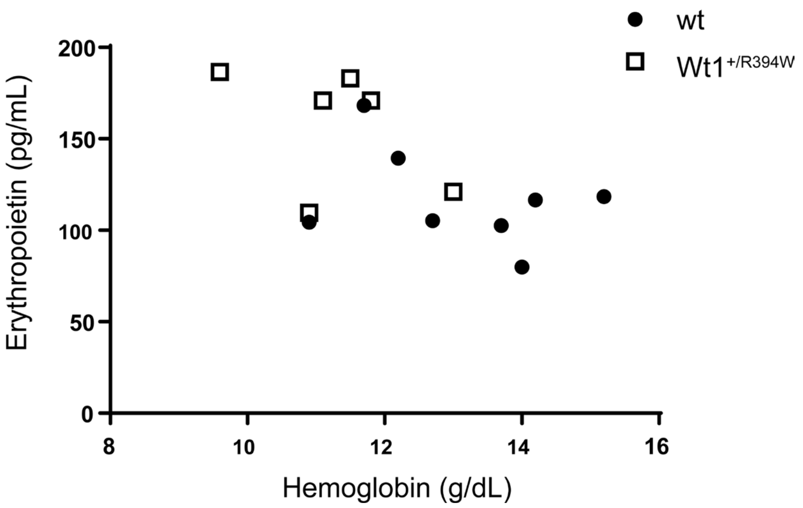

Supplementary Figure 1: Erythropoietin levels correlate with hemoglobin levels similarly in wild type and *Wt1*$^{+/R394W}$ mice. Mouse serum from wild type (wt) and *Wt1*$^{+/R394W}$ mice at the time of sacrifice was evaluated for erythropoietin levels. Hemoglobin (g/dL) is plotted against erythropoietin (pg/mL). Correlation coefficient for wild type mice = 0.174, correlation coefficient for *Wt1*$^{+/R394W}$ = 0.2207.

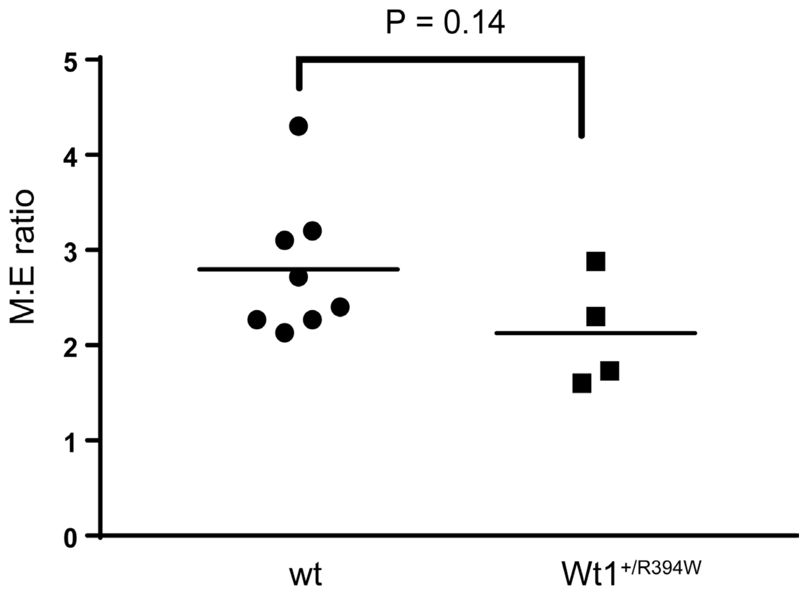

Supplementary Figure 2: Moribund *Wt1*$^{+/R394W}$ mice demonstrate a trend towards decreased M:E ratios. Comparison of myeloid to erythroid (M:E) ratios in bone marrow of moribund *Wt1*$^{+/R394W}$ mice ($n = 4$, mean M:E ratio = 2.13 ± 0.29) compared to age-matched wild type (wt) mice ($n = 8$, mean M:E ratio = 2.8 ± 0.26; $p = 0.14$). Horizontal bars represent the mean values.

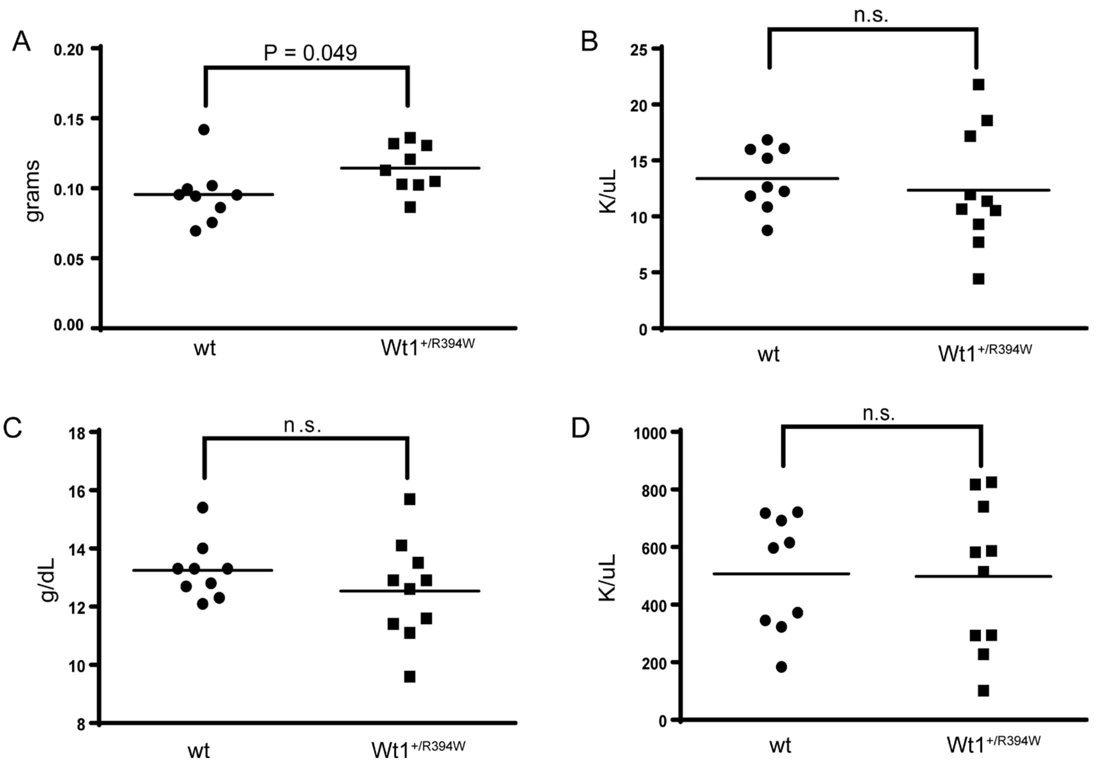

Supplementary Figure 3: Hematologic characteristics of 2-month old pre-disease wild type and *Wt1*$^{+/R394W}$ mice. (**A**) Spleen weights. Wild type (wt) mice had a mean spleen weight of 0.096 ± 0.0069 grams, versus *Wt1*$^{+/R394W}$ mice with a mean spleen weight of 0.114 ± 0.0056 grams (p = 0.049). (**B**) WBC. Wild type mice had a mean WBC of 13.38 ± 0.92 K/uL compared to 12.35 ± 1.67 K/uL for *Wt1*$^{+/R394W}$ mice (p = 0.6) (**C**) Hemoglobin. Mean hemoglobin was 13.24 ± 0.33 g/dL for wt mice and 12.54 ± 0.54 g/dL for *Wt1*$^{+/R394W}$ mice (p = 0.29). (**D**) Platelet counts. Platelet count mean was 507.4 ± 67.4 K/uL for wt mice and 498 ± 81.4 K/uL for *Wt1*$^{+/R394W}$ mice (p = 0.93). Horizontal bars represent the mean values.

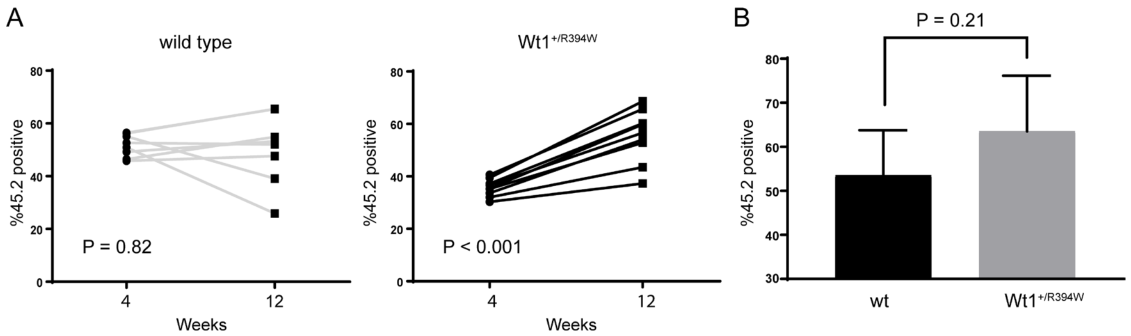

Supplementary Figure 4: Competitive repopulation assay of wild type (wt) versus *Wt1*$^{+/R394W}$ progenitor cells. Transplant recipients received a 1:1 mixture of 45.1 wild type (wt) whole bone marrow cells with either 45.2 wt or 45.2 *Wt1*$^{+/R394W}$ whole bone marrow cells. (**A**) Trend of CD45.2 engraftment in the peripheral blood (PB) of wt (left panel) and *Wt1*$^{+/R394W}$ (right panel) competitive transplant recipients, as measured by flow cytometry at 4 and 12 weeks after transplant. (**B**) Comparison of %CD45.2 engraftment in wt and *Wt1*$^{+/R394W}$ bone marrow as measured by flow cytometry at 16 weeks after transplant. Data is represented as the mean, error bars represent the SEM.

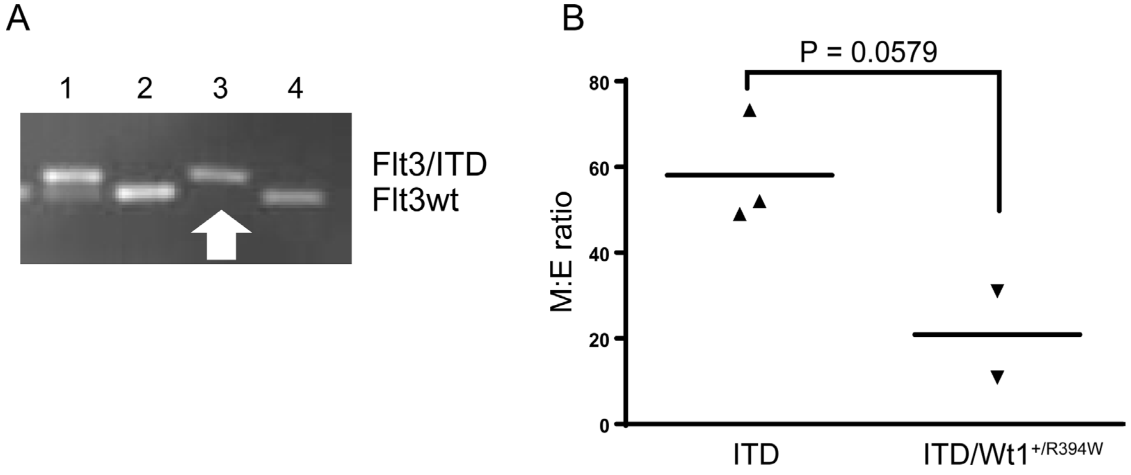

Supplementary Figure 5: Loss of heterozygosity (LOH) of wild type *Flt3* in *Flt3*$^{+/ITD}$ and *Flt3*$^{+/ITD}$/*Wt1*$^{+/R394W}$ bone marrow. (**A**) PCR amplification of *Flt3* in bone marrow DNA demonstrates loss of the wild type allele of *Flt3* (Flt3wt) in a moribund *Flt3*$^{+/ITD}$ mouse in lane 3 and denoted by the white arrow, as compared to the bone marrow of another *Flt3*$^{+/ITD}$ mouse (lane 1) and the bone marrow of two wild type mice (lanes 2 and 4). (**B**) Myeloid to erythroid (M:E) ratios. *Flt3*$^{+/ITD}$ mice (ITD) with LOH of wild type *Flt3* had a mean M:E ratio in the bone marrow of 58.1 ± 7.7, versus *Flt3*$^{+/ITD}$/*Wt*1$^{+/R394W}$ mice (ITD/Wt1$^{+/R394W}$) with LOH of wild type *Flt3*, which had a mean M:E ratio of 20.9 ± 10.1 (p = 0.0579). Horizontal bars represent the mean values.

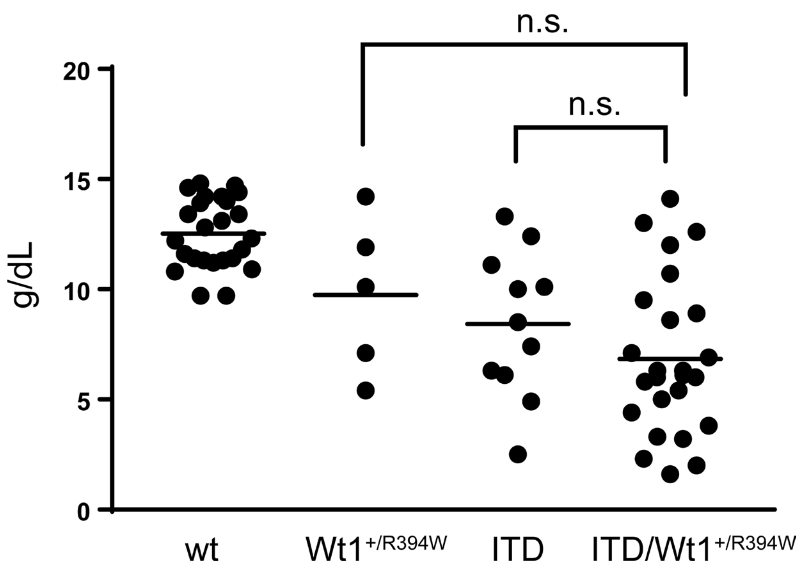

Supplementary Figure 6: Hemoglobin values of moribund mice. Complete blood counts were performed at sacrifice. Horizontal bars represent the mean values. Median hemoglobin values: wild type (wt) 12.6 ± 0.33 g/dL, *Wt1*$^{+/R394W}$ 9.7 ± 1.6 g/dL, ITD 8.4 ± 1 g/dL, and ITD/*Wt1*$^{+/R394W}$ 6.8 ± 0.6 g/dL. *Wt1*$^{+/R394W}$ vs. ITD/*Wt1*$^{+/R394W}$ (p = 0.15), ITD vs. ITD/*Wt1*$^{+/R394W}$ (p = 0. 19).
